# HLA-B*57:01/Carbamazepine-10,11-Epoxide Association Triggers Upregulation of the NFκB and JAK/STAT Pathways

**DOI:** 10.3390/cells12050676

**Published:** 2023-02-21

**Authors:** Funmilola Josephine Haukamp, Zoe Maria Hartmann, Andreas Pich, Joachim Kuhn, Rainer Blasczyk, Florian Stieglitz, Christina Bade-Döding

**Affiliations:** 1 Institute of Transfusion Medicine and Transplant Engineering, Hannover Medical School, Carl-Neuberg-Str. 1, 30625 Hannover, Germany; 2 Institute of Toxicology, Hannover Medical School, Carl-Neuberg-Str. 1, 30625 Hannover, Germany; 3 Core Facility Proteomics, Hannover Medical School, Carl-Neuberg-Str. 1, 30625 Hannover, Germany; 4 Institute for Laboratory and Transfusion Medicine, Heart and Diabetes Center North Rhine-Westphalia, Ruhr University Bochum, Georgstraße 11, 32545 Bad Oeynhausen, Germany

**Keywords:** adverse drug reaction, HLA, carbamazepine hypersensitivity, carbamazepine, carbamazepine-10,11-epoxide, proteome, HLA-B*57:01

## Abstract

Measure of drug-mediated immune reactions that are dependent on the patient’s genotype determine individual medication protocols. Despite extensive clinical trials prior to the license of a specific drug, certain patient-specific immune reactions cannot be reliably predicted. The need for acknowledgement of the actual proteomic state for selected individuals under drug administration becomes obvious. The well-established association between certain HLA molecules and drugs or their metabolites has been analyzed in recent years, yet the polymorphic nature of HLA makes a broad prediction unfeasible. Dependent on the patient’s genotype, carbamazepine (CBZ) hypersensitivities can cause diverse disease symptoms as maculopapular exanthema, drug reaction with eosinophilia and systemic symptoms or the more severe diseases Stevens-Johnson-Syndrome or toxic epidermal necrolysis. Not only the association between HLA-B*15:02 or HLA-A*31:01 but also between HLA-B*57:01 and CBZ administration could be demonstrated. This study aimed to illuminate the mechanism of HLA-B*57:01-mediated CBZ hypersensitivity by full proteome analysis. The main CBZ metabolite EPX introduced drastic proteomic alterations as the induction of inflammatory processes through the upstream kinase ERBB2 and the upregulation of NFκB and JAK/STAT pathway implying a pro-apoptotic, pro-necrotic shift in the cellular response. Anti-inflammatory pathways and associated effector proteins were downregulated. This disequilibrium of pro- and anti-inflammatory processes clearly explain fatal immune reactions following CBZ administration.

## 1. Introduction

The approval of a medical product requires extensive and distinct clinical trials. Yet, the preselected group of volunteers who attend those clinical trials is limited. Every single person has a unique genetic profile affecting the functionality of any cell type of the immune system. It becomes obvious that drug-hypersensitivity reactions in most cases disorganize the adaptive immune system, resulting in severe cellular autoimmune reactions. In the past, these reactions resulted in the mandatory determination of distinct genetic profiles and at worst in the exclusion of patients from the desired medication. It is clear that these scenarios of hypersensitivity reactions following drug treatment represent an unpredictable challenge for the health care system.

Adverse events occur when harmful symptoms arise after administration of a certain drug. If the harm is caused by application of the respective drug, the immunological reaction is termed an adverse drug event; if the drug was applied correctly at normal dosage, the reaction is termed an adverse drug reaction (ADR) [1,2,3,4,5,6]. ADRs usually occur in a dose-dependent and predictable manner and can be explained by the pharmacological toxicity of the drug [1,2,7]. Nevertheless, in 20% of all ADRs, their occurrence seems idiosyncratic; those reactions are termed type B ADRs [1,2,8]. Yet, type B ADRs are often related to the immune system [1]. Since 2002, more and more type B ADRs have been described to be associated with the highly polymorphic human leukocyte antigen (HLA) molecules [9,10,11,12]. HLA molecules are cell surface proteins with a central function in immune surveillance. They present peptides to immune receptors of T and NK cells and, based on the origin of the presented peptide (i.e., self-peptide or pathogen-derived peptides), effector cell responses are prevented or induced [13,14]. The presentation of a diversity of peptides derived from different origins is unique in the ligand/receptor biology, since every peptide bound to an HLA molecule results in structural and biophysical alteration of the peptide-HLA complex. Therefore, it becomes clear that every subtle variation in the HLA molecule might facilitate binding and presentation of peptides that have not undergone selection by the thymus; the biological consequences are autoimmune-like reactions [15,16].

The anticonvulsant carbamazepine (CBZ) is widely used to treat various neurological diseases such as epilepsy, bipolar disorders or schizophrenia. However, CBZ administration can cause cutaneous type B ADRs in certain patients. These reactions have been described to be associated with the human leukocyte antigen (HLA) class I genotypes HLA-B*15:02 and HLA-A*31:01 [11,17].

Depending on the patient’s genotype, CBZ-induced ADRs are characterized by differential disease phenotypes. The symptoms range from mild skin rash such as maculopapular exanthema (MPE) and drug reaction with eosinophilia and systemic symptoms (DRESS) to more severe and potentially fatal Stevens-Johnson syndrome (SJS) and toxic epidermal necrolysis (TEN) [18,19]. It has been shown that the more severe SJS and TEN occur mainly in HLA-B*15:02+ patients, whereas MPE and DRESS following CBZ-treatment more likely arise in HLA-A*31:01+ patients [11,20].

Positive and negative predictive values indicate that the clinical picture of HLA-associated ADRs cannot be explained exclusively by the presence of a certain HLA allele [21], hence other factors have to be taken into account [22]. We could demonstrate that CBZ treatment in soluble HLA-A*31:01-expressing cells and EPX treatment in soluble HLA-B*15:02-expressing cells result in different alterations in the cellular proteome that might contribute to the explanation of distinct clinical pictures of the diseases [23].

Recently, a further association of CBZ-induced ADRs has been described. The allele HLA-B*57:01 is unambiguously associated with SJS/TEN following treatment with CBZ in Europeans [24]: The analysis included 28 European patients with CBZ-induced SJS, SJS/TEN-overlap or TEN, 11 of them were carrying HLA-B*57:01 (39.29%), whereas the frequency of this allele was 6.69% in European general population controls. The onset of SJS/TEN following drug application should be close-meshed monitored, an algorithm of drug causality (ALDEN) has been adjusted to provide safe diagnosis [25]. The allele HLA-B*57:01 is originally known to be strongly associated with hypersensitivity to the antiretroviral drug abacavir (ABC) [26,27]. ABC-induced ADRs for HLA-B*57:01+patients vary from fever, fatigue, gastrointestinal symptoms to severe multiorgan failure. For this disease pattern, autologous cytotoxic T cells that attack in a manner like an autoimmune reaction, the body itself could be verified to be responsible [28]. Illing et al. [29] could impressively show that ABC occupies the peptide binding region (PBR) of HLB-B*57:01 resulting in a conformational change of the HLA molecule and therefore, in CD8+-mediated foreign recognition of the self-HLA-B*57:01 molecules bound to a foreign peptide. Since then, this finding provides the gold standard for understanding HLA-mediated ADRs [29]. Patients with susceptible HLA variants have not been permitted to take certain drugs. However, more and more clinical studies recently have demonstrated that drug-tolerant patients exist [21]; namely, patients with a certain HLA type who still could receive the questionable drug even though no immunological reactions occurred. This seems difficult to believe since drug binding and, subsequently, loading of a different peptide repertoire into the peptide binding region of the respective HLA molecule should still occur. However, in some cases a slight alteration in the amino acid sequence of bound peptides is not sufficient to trigger T cell responses. This would lead to maintained T cell tolerance in certain patients [30,31]. These drug-tolerant patients could receive the respective drug regardless of their HLA type. Appreciation of this phenomenon can certainly take place by a real-time view on the proteomic content of cell with the susceptible HLA type and the respective drug. Modern proteomics provide deep insight into the health status, biological and functional opportunities of a cell, and would therefore provide a stage to monitor pharmacovigilance. The observation of an association between HLA-B*57:01 and CBZ-mediated ADRs is in this respect remarkable, since it further emphasizes that CBZ hypersensitivity seems to be associated with several HLA alleles that differ structurally. CBZ hypersensitivity was formerly an immunological reaction that targeted patients with HLA-A*31:01 or B*15:02 following drug administration. We were recently able to demonstrate why CBZ hypersensitivity features completely different clinical pictures depending on the HLA type. Although HLA-A*31:01 would bind CBZ, B*15:02 would preferably bind EPX [32]. Both small-molecule (drug)/protein (HLA) complexes would alter the HLA-specific peptidome by the occupation of the PBR, yet the T cell response manifested by the HLA-specific clinical picture would differ significantly. Clarification for the relation between HLA molecule and drug could in this case be delivered by complete proteome analysis [23].

The aim of this work is to give a first insight into the complex molecular basics of HLA-B*57:01-associated CBZ-mediated ADRs. This knowledge will contribute to a comprehensive understanding of the mechanisms of CBZ hypersensitivities that seem to represent disparate diseases. To achieve sufficiency in genetically based CBZ immune effects, we performed full proteome analysis of HLA-B*57:01 expressing cells in response to CBZ or EPX treatment. Understanding the pharmacological and biological basis of distinct genetic profiles and drug interplay will guide towards personalized and safe medication. 

## 2. Materials and Methods

### 2.1. Detection of CBZ and EPX Bound to sHLA-B*57:01 Molecules

The human B-lymphoblastoid cell line *LCL721.221* (LGG promochem, Wesel, Germany) has been transduced with a lentiviral construct encoding for HLA-B*57:01 Exon 1–4, as previously described [33]. *LCL721.221* cells expressing sHLA-B*57:01 molecules were cultured in RPMI 1640 (Lonza, Basel, Switzerland) supplemented with 10% fetal calf serum (FCS, Lonza), 2 mM L-glutamine (c. c. pro, Oberdorla, Germany), 100 U/mL penicillin, and 100 µg/mL streptomycin (c. c. pro) at 37 °C and 5% CO_2_ in the presence of 25 µg/mL CBZ or EPX and cell culture supernatants were collected twice a week. Affinity purification of sHLA-B*57:01 molecules post drug treatment was performed and protein concentration was calculated by Bicinchoninic Acid Assay (BCA) Protein Quantitation Kit (Interchim, San Diego, CA, USA). 150 ng purified drug-treated sHLA-B*57:01 molecules were applied to mass spectrometric drug quantification in solution as previously described [32].

### 2.2. Detection of CBZ- or EPX-Induced Modifications of the LCL721.221/HLA-B*57:01 Proteome

Proteome analysis was performed with 1 × 10^6^ untreated, CBZ- or EPX-treated *LCL721.221* and LCL721.221/sHLA-B*57:01 cells. Parental and sHLA-B*57:01-expressing *LCL721.221* cells are not able to metabolize CBZ to EPX; this enables the analysis of CBZ and EPX treatment orthogonally. Cells were cultured in addition of 25 µg/mL CBZ or EPX for 48 h. After 24 h, drug addition was repeated. Cell harvest in RIPA lysis was performed as previously described [34] and calculation of protein concentration was performed by Bicinchoninic Acid Assay (BCA) Protein Quantitation Kit (Interchim, San Diego, CA, USA). Sample preparation, protein digestion and MS analysis was performed as previously described [23,35].

### 2.3. Data Analysis

The MaxQuant software (version 1.6.3.3; [36]) was used to search the obtained spectra against the Swiss-Prot reviewed UniProtKB database (version 01/2021, 20,395 entries; [37]). Propionamid of cysteine was set as fixed modification and oxidation of methionine, N-terminal acetylation, deamidation of glutamine, and asparagine were set as variable modifications The data were processed with the Perseus software (version 1.6.2.3; [38]). In brief, proteins that resemble a possible contamination, only identified by sight or were reversed were excluded from further analysis as well as proteins that were not measured in all replicates. To exclude potential effects on protein abundance caused by transduction with sHLA-B*57:01, the proteome of untreated LCL721.221/sHLA-B*57:01 and parental *LCL721.221* cells were subtracted from the corresponding CBZ- or EPX-treated cells. Visualization was performed with R [39]. In particular, the R packages complex heat map [40] and ggplot2 [41] were used. The heat map was generated by including the proteins that were positively tested in a Benjamini Hochberg FDR-based ANOVA. The Ingenuity Pathway Analysis software was used to perform an upstream analysis of significantly regulated proteins (IPA, QIAGEN Inc., https://www.qiagenbio-informatics.com/products/ingenuity-pathway-analysis (accessed on 24 November 2022)). Gene ontology analysis was performed with the GSEA software [42,43]. The mass spectrometry proteomics data were deposited to the ProteomeXchange Consortium via the PRIDE [44] partner repository with the dataset identifier PXD037502.

## 3. Results

### 3.1. CBZ and EPX Bind to sHLA-B*57:01

To verify binding of CBZ or EPX to sHLA-B*57:01 molecules, sHLA-B*57:01 expressing cells were cultured in the presence of 25 µg/mL CBZ or EPX, and sHLA-B*57:01 containing cell culture supernatant was collected twice a week. Functional sHLA-B*57:01 molecules were affinity purified by an *NHS* column coupled to the mAb W6/32 and protein concentration was determined as previously described [45].

**Figure 1:**
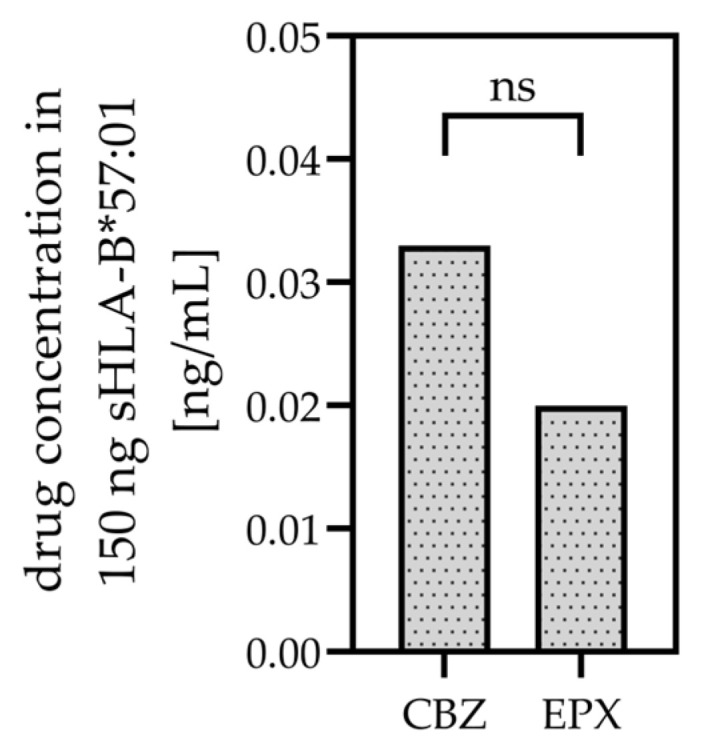
CBZ and EPX concentration on sHLA-B*57:01 molecules post CBZ or EPX treatment. 150 ng affinity purified sHLA-B*57:01 molecules post drug treatment were measured in four replicates (*n* = 4) by UPLC-MS/MS.

150 ng CBZ- or EPX-treated sHLA-B*57:01 molecules were applied to UPLC-MS/MS analysis for detection of CBZ or EPX in solution [32]. CBZ as well as EPX could be verified to bind to sHLA-B*57:01 molecules. In the solution with 150 ng CBZ/sHLA-B*57:01 molecules 0.033 ng/mL CBZ could be detected and in the EPX-containing sHLA-B*57:01 solution 0.020 ng/mL EPX could be detected (Figure 1). 

### 3.2. Quantitative Proteomic Analysis after CBZ and EPX Treatment

**Figure 2:**
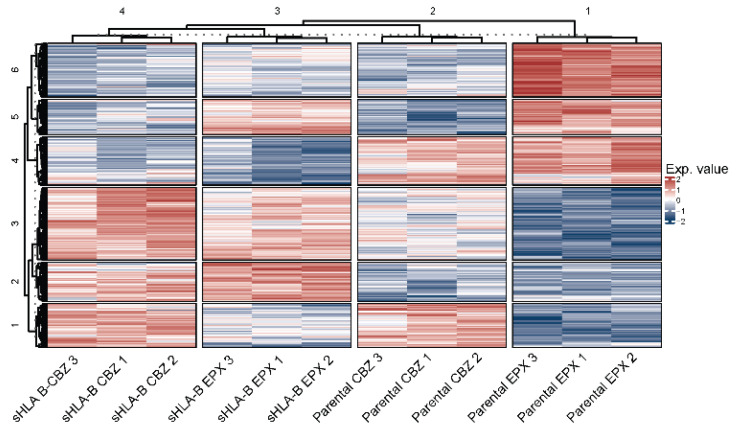
Proteome analysis LCL721.221/sHLA-B*57:01 cells or parental *LCL721.221* cells treated with either CBZ or EPX. Heat map of proteins significantly changed (*p* < 0.05) after FDR-based ANOVA testing.

The cellular proteomes of parental *LCL721.221* cells and LCL721.221/sHLA-B*57:01 cells were analyzed in an LFQ-based approach. For comparison of CBZ or EPX treatment of HLA-B*57:01 expression and parental *LCL721.221* cells, the proteomic content of untreated *LCL721.221* and LCL721.221/sHLA-B*57:01 cells was subtracted from the drug-treated proteome abundances. In total, 4519 proteins could be identified. To exclude proteins that were induced through transduction with the HLA-B*57:01 allele, only proteins were included in the analysis that were measured in all replicates without imputation. After filtering, 2713 proteins were feasible for further research. By subtracting the untreated LCL721.221/sHLA-B*57:01 and parental *LCL721.221* cells from the corresponding CBZ- or EPX-treated cells, possible effects on the proteome introduced by the transduction were excluded. The data were analyzed for their examinability through dimensionality reduction with t-SNE, and clustering of the different treatments confirmed that the data were feasible for further analysis. Distinct clustering also occurred in the heat map analysis (Figure 2). 

### 3.3. EPX Treatment Induced a Strong Reaction in the Proteome of LCL721.221/sHLA-B*57:01 Cells

**Figure 3:**
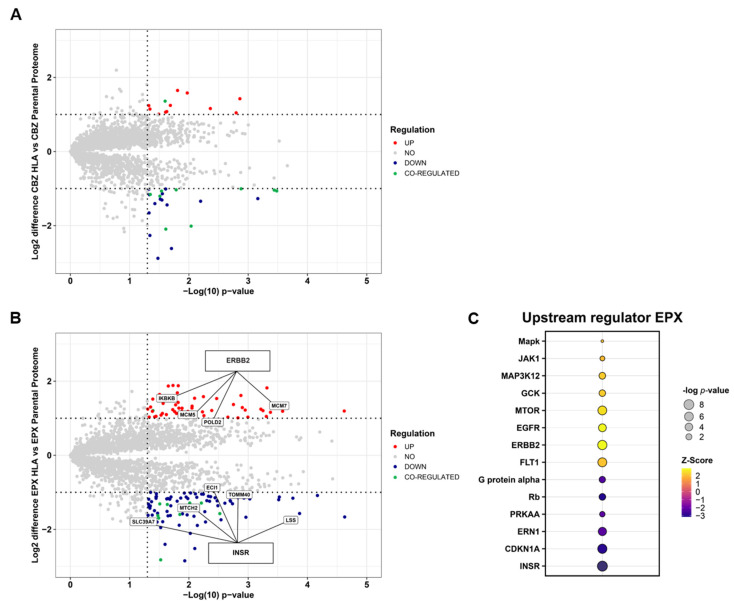
Analysis of proteomic changes and predicted upstream regulators after treatment of LCL721.221/sHLA-B*57:01 and parental *LCL721.221* cells with CBZ or EPX. (**A**) Volcano plot of protein expression in LCL721.221/sHLA-B*57:01 vs. parental *LCL721.221* cells after CBZ treatment. (**B**) Volcano plot of protein expression in LCL721.221/sHLA-B*57:01 vs. parental *LCL721.221* cells after EPX treatment. Highlighted proteins were predicted to be responsive to the predicted upstream regulator ERBB2 or ISNR after EPX treatment and are altered significantly with at least a two-fold change in expression. Horizontal dotted line indicates two-fold difference, vertical line *p*-value threshold of *p* < 0.05, blue dots: significant two-fold downregulation, red dots: Two-fold significant upregulation. Up- and downregulated proteins that overlap between CBZ- and EPX-treated cells are depicted in green. (**C**) Predicted upstream regulators by IPA™.

CBZ treatment induced a significant change of abundance (*p* < 0.05) of 335 proteins with only 35 changes more than 2-fold in LCL721.221/sHLA-B*57:01 cells when compared to parental cells (Figure 3A). However, EPX treatment led to 776 significantly changed proteins and 134 proteins with a difference greater than 2-fold (Figure 3B). Furthermore, we found ten proteins showing an overlapped regulation between CBZ- and EPX-treated regulation with one protein being co-upregulated and nine proteins being co-downregulated (Figure 3A,B and Figure S2). An upstream analysis with the IPA software was performed to find central regulators responsible for the change in abundance. For CBZ treatment, the serine/threonine kinase IKBKE was suggested as the only upstream kinase that is activated (*p*-value 0.0457; Z-Score 2.0). At the same time, treatment with EPX led to the regulation of 14 kinases, with the receptor tyrosine kinase ERBB2 as the most activated, and the insulin receptor INSR predicted to be the most inactivated kinase. According to IPA upstream analysis, ERBB2 is responsible for the upregulation of IKBKB, MCM5, POLD2 and MCM7. In contrast, INSR leads to the downregulation of SLC39A7, MTCH2, ECI1, TOMM40 and LSS. Other activated indicated upstream regulators were part of the MAPK protein family or involved in the MAPK signaling cascade. In comparison, downregulated upstream regulators were predicted to be G Protein alpha, Rb, PRKAA, ERN1 and CDKN1A (Figure 3C).

**Figure 4:**
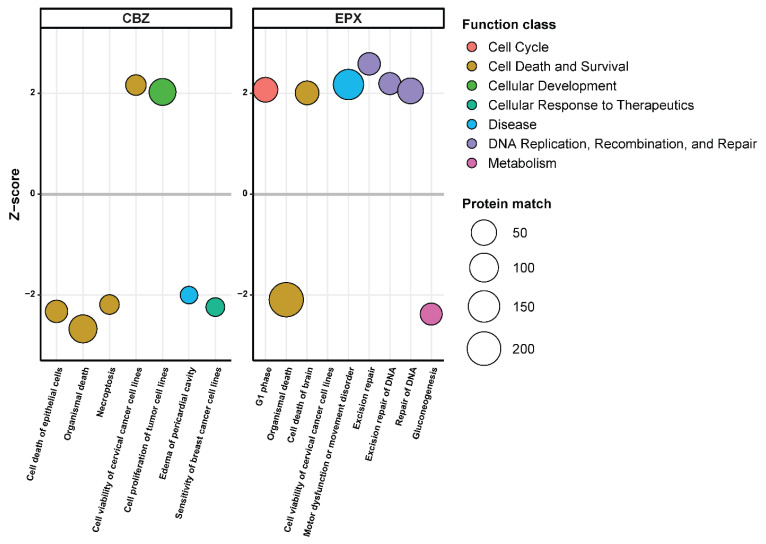
IPA disease and function analysis of proteomic changes induced by EPX or CBZ treatment in LCL721.221/mHLA-B*57:01 cells vs. parental *LCL721.221* cells. Only functions are depicted that were predicted to be up- or downregulated (Z-score > 2 or <−2) by the IPA software.

Further analysis of function classes of significantly regulated proteins via the IPA software showed that EPX treatment induced expression of proteins involved in “DNA Replication, Recombination, and Repair”. Furthermore, cell cycle-related proteins were found to be upregulated (Figure 4). Downregulated proteins were involved in “organismal death” and “glycogenesis”. CBZ treatment led to the downregulation of proteins involved in “organismal death”, “necroptosis”, and “cell death of epithelial cells” whereas proteins involved in “cell proliferation of Tumor cell lines” were upregulated. 

**Figure 5:**
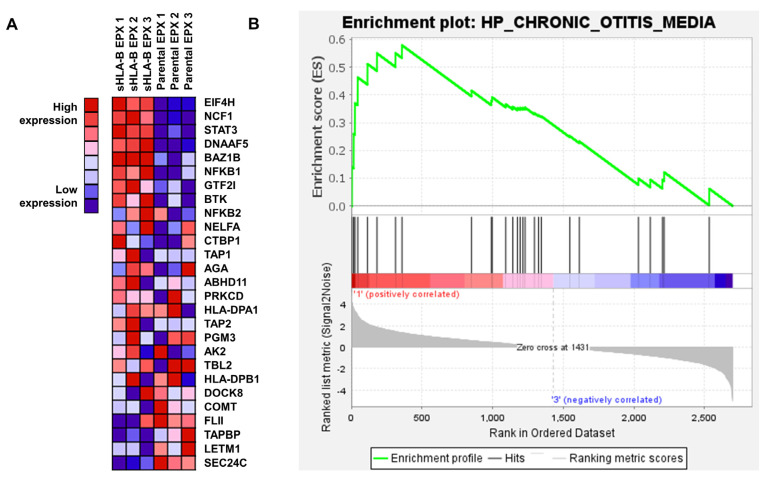
GSEA global enrichment analysis of proteomic changes induced by EPX treatment in LCL721.221/mHLA-B*57:01 cells vs. parental cells. (**A**) Heatmap of regulated proteins in HLA-B*57:01 expressing and parental *LCL721.221* cells following EPX treatment. (**B**) GSEA enrichment plot HP_CHRONIC_OTTIS_MEDIA.

Global GSEA enrichment analysis showed enrichment (Enrichment score: 0.58) in protein expression involved in an inflammatory pathway (“HP_CHRONIC_OTITIS_MEDIA”) in LCL721.221/sHLA-B*57:01 cells that were treated with EPX compared to parental *LCL721.221* cells treated with EPX. ELF4H, NCE1, STAT3, DNAAF5, RAZIB, NFKB1 were upregulated following EPX treatment in LCL721.221/sHLA-B*57:01 cells and were downregulated in parental cells after EPX treatment (Figure 5).

EPX treatment induced the regulation of 14 pathways in the 25 most significant pathways predicted by the IPA software whereas CBZ treatment induced the regulation of 10 pathways. The most activated pathway after EPX treatment was predicted to be the “Necroptosis Signaling Pathway”, and “2-ketoglutarate Dehydrogenase Complex” was predicted to be most downregulated. CBZ treatment induced the most robust activation of the “G2/M DNA Damage Checkpoint Regulation” and inhibited “ELF2 Signaling” (Figure S1). 

## 4. Discussion

Recent studies have demonstrated that besides HLA-A*31:01 and HLA-B*15:02, HLA-B*57:01 is also strongly linked to CBZ-induced ADRs. Although CBZ administration in HLA-A*31:01^+^ patients causes diseases such as MPE and DRESS, CBZ administration in HLA-B*57:01^+^ Europeans resulted in SJS/TEN disease phenotypes [24] as observed for HLA-B*15:02^+^ patients [11]. SJS and TEN manifest severe life-threatening cutaneous and mucosal necrosis and have to be treated by specialized burns units [46]. When SJS/TEN emerge as HLA-mediated ADRs that involve T cell recognition of foreign peptide/HLA-complexes, the withdrawal of the drug should assure recovery of the clinical state. SJS/TEN is such an intense impairment of the affected skin that recovery is rarely possible. Therefore, prevention of such an adverse condition is mandatory in pharmacovigilance management strategies. Although the prophylaxis of HLA-mediated ADRs is not feasible and individual patient cases are often underreported due to the polymorphic character of HLA molecules, the need for conscientious analysis of HLA-mediated ADRs immediately following their establishment should be obvious. HLA molecules exhibit unique properties in the immune system. Host HLA molecules bind foreign antigens. This exceptional co-recognition requires exquisite specificity and genetical restriction for the host T cells [13]. HLA diversity and corresponding T cell diversity restrict a comprehensive screening of patient cohorts in phase I, II and III studies [47,48] where pharmacokinetics and pharmacodynamics prior to admission of a drug are tested. In the era of fast and sophisticated methods to view into the cellular content, proteomics are the instrument for understanding and long-term prevention of HLA-mediated ADRs. HLA-restricted peptidomics and T cell analysis deliver indisputable answers to understand immune compatibility, but in some cases the host T cells fail to recognize the presented peptide/drug/HLA ligand of host origin. Understanding indistinct intracellular activities as metabolism, cytokine expression, and downregulation of certain proteins in drug-tolerant patients would certainly be beneficial for drug-sensitive patients with a susceptible HLA type. Utilizing proteomics as a mirror into cellular events should support this objective.

In the present study, we aimed to illuminate the underlying mechanism of HLA-B*57:01-mediated hypersensitivity to CBZ by full proteome analysis of CBZ- or EPX-treated LCL721.221/sHLA-B*57:01 cells. We chose the lymphoblastoid *LCL721.221* cells, because these cells are not able to metabolize CBZ to EPX. The metabolization of CBZ to EPX occurs exclusively in hepatocytes and is catalyzed by cytochrome P450 enzymes [49]. Thus, the impact of CBZ and EPX treatment on the cellular proteome of *LCL721.221* cells can be analyzed orthogonally.

Prior to proteome analysis, the specificity of drug-HLA interaction was determined via UPLC-MS/MS analysis. The selection of CBZ or the metabolite EPX by the respective HLA molecule is decisive for the fate of the HLA-expressing cell as previously described [32]. We could previously demonstrate that CBZ binds exclusively to HLA-A*31:01, leading to severe skin lesions and that the exclusive interaction between EPX-HLA-B*15:02 and not CBZ-HLA-B*15:02 [32] led to life-threatening SJS/TEN diseases. The present study showed that HLA-mediated ADRs have to be meticulously analyzed to comprehensively understand their clinical outcome. In this paper, we can show that both drug conditions CBZ and EPX are able to engage with HLA-B*57:01. The main question occurs if both or one drug condition would, in cooperation with HLA-B*57:01, impact the cellular content of the antigen-presenting cells and possibly their microenvironment. Therefore, *LCL721.221* cells have been transduced with sHLA-B*57:01 and exposed to the respective drug. *LCL721.221* cells are not able to metabolize CBZ to EPX and are thus a meaningful instrument to answer the question.

LCL721.221/sHLA-B*57:01 cells were treated with 25 µg/mL CBZ or EPX, respectively, and cell lysates were applied to full proteome analysis. By subtracting the proteomic changes that were introduced through transduction of the cells with the HLA-B*57:01 allele, we were able to observe the independent effects that occurred due to the interplay of CBZ or EPX with the HLA-B*57:01 molecule. 

We found that EPX treatment induced significant changes in the proteome of LCL721.221/sHLA-B*57:01 cells. In contrast, CBZ treatment resulted in minimal changes in the proteome of LCL721.221/sHLA-B*57:01 cells. CBZ treatment of LCL721.221/sHLA-B*57:01 cells led to only 35 significantly regulated proteins whereas EPX treatment of the cells resulted in 134 significantly regulated proteins. Only a slight overlap of ten significantly regulated proteins could be detected in both CBZ- and EPX-treated cells (Figure 3A and Figure S2). Upstream regulator analysis via IPA revealed just one activated upstream regulator, the serine/threonine kinase IKBKE, responsible for the change in protein abundance of CBZ-treated cells. In contrast, 14 kinases were detected as regulated in EPX-treated cells (Figure 3C). Although UPLC-MS/MS analysis revealed equal binding of CBZ and EPX to sHLA-B*57:01 molecules (Figure 1), the CBZ-induced changes of the cellular proteome of LCL721.221/sHLA-B*57:01 seem to be marginal when compared to EPX-induced changes. 

Following EPX treatment, the receptor tyrosine kinase ERBB2 could be described to be the most activated upstream regulator (Figure 3). ERBB2 is mainly involved in inflammatory and growth-associated processes [50]. Consequently, proteins that were predicted to be influenced and significantly two-fold upregulated were IKBKB, MCM5, MCM7, and POLD2. IKBKB is described to activate NFκB that is involved in inflammatory, pro-apoptotic and necrotic processes [51]. Additionally, NFκB has been found to be significantly enriched in the GSEA enrichment analysis in an overall inflammatory process (Figure 5). MCM5 and MCM7 are involved in DNA replication and are responsive to cytokine-induced gene transcription. MCM5 has been shown to be central for STAT1-mediated gene transcription [52]. In line with this, JAK1 has also been predicted to be activated (Figure 3). The JAK/STAT pathway plays a central role in reaction to external inflammatory stimuli [53]. Consistent with this finding, STAT5 upregulation has also been described for HLA-B*15:02 after EPX treatment [23]. The comparison of proteomic profiling of cells with intracellular small molecule/protein engagement [23] features clearly that EPX/HLA engagement triggers the upregulation of inflammatory pathways. The sudden upregulation of proteins that are described to be part of signal transduction pathways and thus triggers of autoimmune reactions through effector cell activation could not be predicted by conventional methods. We further describe the upregulation of POLD2, an enzyme that is involved in DNA repair processes and preserving DNA integrity [54]. POLD2 could recently be uncovered as a tumor suppressor [55] and prognostic biomarker in distinct cancers [56]. In coherence with POLD2 upregulation was the finding that more than 50 proteins involved in DNA repair, replication and recombination were regulated in response to EPX treatment and DNA regulatory processes were predicted to be activated (Figure 4). Moreover, FLT1 and EGFR were also indicated as activated. Both are known for their potential to induce apoptosis through either NFκB (FLT1) [57] or STAT3 (EGFR) activation [58]. FLT1 could be described as a therapeutic target in inflammatory events [59] whereas EGFR is known as a key regulator in cell division and cancer development [60]. In conclusion, the engagement of EPX/HLA-B*57:01 induces inflammatory, pro-apoptotic and necrotic processes in *LCL721.221* cells when compared to parental *LCL721.221* cells. These findings seem to be consistent with and might be a coherent explanation of the disease phenotype of SJS/TEN in HLA-B*57:01^+^ patients that is associated with keratinocyte death, cutaneous blistering and epidermal detachment [61].

In coherence with the upregulation of proinflammatory proteins, INSR could be observed to be inactivated after EPX treatment (Figure 3). INSR has been described as inhibiting inflammatory and cytokine-mediated processes when overexpressed [62]. These data illustrate the dignified intracellular cooperation between signal transduction proteins and the unpredictable interference of drug/HLA complexes. In addition, CDKN1A is predicted to be inhibited (Figure 3). Although CDKN1A is an inhibitor for cell proliferation in B cells, CDKN1A acts as an activator of proliferation and is closely regulated through Caspase-3 mediated degradation [63]. CDKN1A downregulation might suggest Caspase-3 activation and cleavage of CDKN1A. 

The “Necroptosis Signaling Pathway” was detected to be the most activated pathway following EPX treatment of LCL721.221/sHLA-B*57:01 cells. Cell death through necroptosis is a form of programmed necrosis that is mediated by pattern recognition receptors (PRR) and diverse cytokines. Necroptosis of cells results in the secretion of damage-related pattern molecules (DAMPs) and, subsequently, an inflammatory immune response [64,65]. 

Our observations indicate an unbalance of pro- and anti-inflammatory processes through up- or rather downregulation of certain proteins that might lead to an excessive immune reaction in the affected patients with the susceptible HLA allele that is mainly caused by EPX.

Taken together, although EPX/HLA-B*57:01 cooperation introduced the described drastic changes in the proteome, alterations through CBZ/HLA-B*57:01 cooperation were only marginal. It seems obvious that, similar to CBZ-induced hypersensitivity in HLA-B*15:02^+^ patients, EPX is the main driver for the SJS/TEN phenotype in HLA-B*57:01 patients. The engagement of EPX and the HLA molecule seems to perturb the intracellular processing of healthy cells and produces a stress response resulting in DNA damage and consequently, extensive inflammation. The possibility to study the effect of EPX/HLA and CBZ/HLA orthogonally in the present study offers the potential to appreciate the different clinical outcomes of HLA-mediated CBZ hypersensitivity. The metabolization of CBZ to EPX occurs in the cytochrom P450 system in the liver. Although CBZ is metabolized to EPX, the balance between both drugs shifts towards an excess of EPX, the inflammatory life-threatening condition of concerned patients therefore becomes more severe and might shift from the initiation of SJS to TEN. TEN is a serious and fatal condition for which the outcome is in >50% of affected patients lethal or at least leads to incurable long-term harm.

To embed these findings into the biological context of systemic inflammation, the key processes that might drive the hypersensitivity reaction are pathways that were found to be upregulated, indicating a beginning of cell death in HLA-B*57:01 transduced cells, for example the strong activation of necroptosis pathway after EPX treatment (Figure S1). A recent study of the hypersensitivity reaction in HLA-B*15:02^+^ patients revealed that the presence of CD4^+^CD25^+^CD127loCD39^+^ Treg that can reduce the presence of extracellular ATP by degrading it via CD39 and CD73 to adenosine determines the conversion from a non-responder to a responder [66]. By taking this study into account, we hypothesize that releasing intracellular ATP into the extracellular matrix facilitated by inflammatory processes induced by EPX is the initial step towards a systemic inflammation when not enough CD4^+^CD25^+^CD127loCD39^+^ Treg are present to reduce the effect of extracellular ATP and subsequent IFNγ production. 

## 5. Conclusions

The future of pharmacological appreciation of drug and medical safety relies on the comprehension of the functional consequences of individual immunogenetics.

## Data Availability

The mass spectrometry proteomics data have been deposited to the ProteomeXchange Consortium via the PRIDE partner repository with the dataset identifier PXD037502.

## Supplementary Materials

The following supporting information can be downloaded at: https://www.mdpi.com/article/10.3390/cells12050676/s1, Figure S1: Ingenuity pathway analysis of proteomic changes induced by (A) EPX or (B) CBZ treatment in *LCL721.221/mHLA-B*57:01* cells vs. parental cells. Figure S2: Analysis of proteomic changes and predicted upstream regulators after treatment of *LCL721.221/sHLA-B*57:01* and parental *LCL721.221* cells with CBZ or EPX. Volcano plot of protein expression in *LCL721.221/sHLA-B*57:01* vs. parental *LCL721.221* cells after CBZ (A) or EPX treatment (B). Labeled proteins overlap between both treatment conditions.

## References

[1] Simper G., Celik A.A., Kunze-Schumacher R.B.H., Blasczyk R., Bade-Döding C. (2017). Physiology and Pathology of Drug Hypersensitivity: Role of Human Leukocyte Antigens.

[2] Hò G.-G.T., Hiemisch W., Pich A., Matern M., Gräser L.S., Blasczyk R., Bade-Doeding C., Simper G.S. (2021). Small Molecule/HLA Complexes Alter the Cellular Proteomic Content.

[3] Nebeker J.R., Barach P., Samore M. (2004). Clarifying adverse drug events: A clinician’s guide to terminology, documentation, and reporting. Ann. Intern. Med..

[4] Gurwitz J.H., Field T.S., Avorn J., McCormick D., Jain S., Eckler M., Benser M., Edmondson A.C., Bates D.W. (2000). Incidence and preventability of adverse drug events in nursing homes. Am. J. Med..

[5] Bates D.W., Cullen D.J., Laird N., Petersen L.A., Small S.D., Servi D., Laffel G., Sweitzer B., Shea B.F., Hallisey R. (1995). Incidence of adverse drug events and potential adverse drug events. Implications for prevention. ADE Prevention Study Group. JAMA.

[6] Edwards I.R., Aronson J.K. (2000). Adverse drug reactions: Definitions, diagnosis, and management. Lancet.

[7] Rawlins M.D. (1981). Clinical pharmacology. Adverse reactions to drugs. Br. Med. J. (Clin. Res. Ed.).

[8] Pichler W.J., Hausmann O. (2016). Classification of Drug Hypersensitivity into Allergic, p-i, and Pseudo-Allergic Forms. Int. Arch. Allergy Immunol..

[9] Hetherington S., Hughes A.R., Mosteller M., Shortino D., Baker K.L., Spreen W., Lai E., Davies K., Handley A., Dow D.J. (2002). Genetic variations in HLA-B region and hypersensitivity reactions to abacavir. Lancet.

[10] Mallal S., Nolan D., Witt C., Masel G., Martin A., Moore C., Sayer D., Castley A., Mamotte C., Maxwell D. (2002). Association between presence of HLA-B*5701, HLA-DR7, and HLA-DQ3 and hypersensitivity to HIV-1 reverse-transcriptase inhibitor abacavir. Lancet.

[11] Chung W.H., Hung S.-I., Hong H.-S., Hsih M.-S., Yang L.-C., Ho H.-C., Wu J.-Y., Chen Y.-T. (2004). Medical genetics: A marker for Stevens-Johnson syndrome. Nature.

[12] Deshpande P., Hertzman R.J., Palubinsky A.M., Giles J.B., Karnes J.H., Gibson A., Phillips E.J. (2021). Immunopharmacogenomics: Mechanisms of HLA-Associated Drug Reactions. Clin. Pharmacol. Ther..

[13] Zinkernagel R.M., Doherty P.C. (1974). Restriction of in vitro T cell-mediated cytotoxicity in lymphocytic choriomeningitis within a syngeneic or semiallogeneic system. Nature.

[14] Neefjes J., Jongsma M.L.M., Paul P., Bakke O. (2011). Towards a systems understanding of MHC class I and MHC class II antigen presentation. Nat. Rev. Immunol..

[15] Falk K., Rötzschke O., Stevanović S., Jung G., Rammensee H.-G. (2006). Allele-specific motifs revealed by sequencing of self-peptides eluted from MHC molecules. 1991. J. Immunol..

[16] Wieczorek M., Abualrous E.T., Sticht J., Álvaro-Benito M., Stolzenberg S., Noé F., Freund C. (2017). Major Histocompatibility Complex (MHC) Class I and MHC Class II Proteins: Conformational Plasticity in Antigen Presentation. Front. Immunol..

[17] McCormack M., Alfirevic A., Bourgeois S., Farrell J.J., Kasperavičiūtė D., Carrington M., Sills G.J., Marson T., Jia X., De Bakker P.I. (2011). HLA-A*3101 and Carbamazepine-Induced Hypersensitivity Reactions in Europeans. N. Engl. J. Med..

[18] Hari Y., Frutig-Schnyder K., Hurni M., Yawalkar N., Zanni M.P., Schnyder B., Kappeler A., Von Greyerz S., Braathen L.R., Pichler W.J. (2001). T cell involvement in cutaneous drug eruptions. Clin. Exp. Allergy.

[19] Nassif A., Bensussan A., Boumsell L., Deniaud A., Moslehi H., Wolkenstein P., Bagot M., Roujeau J.-C. (2004). Toxic epidermal necrolysis: Effector cells are drug-specific cytotoxic T cells. J. Allergy Clin. Immunol..

[20] Yip V.L.M., Pirmohamed M. (2017). The HLA-A*31:01 allele: Influence on carbamazepine treatment. Pharm. Pers. Med..

[21] Li Y., Deshpande P., Hertzman R.J., Palubinsky A.M., Gibson A., Phillips E.J. (2021). Genomic Risk Factors Driving Immune-Mediated Delayed Drug Hypersensitivity Reactions. Front. Genet..

[22] Hertzman R.J., Deshpande P., Gibson A., Phillips E.J. (2021). Role of pharmacogenomics in T-cell hypersensitivity drug reactions. Curr. Opin. Allergy Clin. Immunol..

[23] Simper G.S., Gräser L.S., Celik A.A., Kuhn J., Kunze-Schumacher H., Hò G.-G.T., Blasczyk R., Pich A., Bade-Doeding C. (2019). The Mechanistic Differences in HLA-Associated Carbamazepine Hypersensitivity. Pharmaceutics.

[24] Mockenhaupt M., Wang C., Hung S., Sekula P., Schmidt A.H., Pan R., Chen C., Dunant A., Le Gouvello S., Schumacher M. (2019). *HLA-B*57:01* confers genetic susceptibility to carbamazepine-induced SJS/TEN in Europeans. Allergy.

[25] Sassolas B., Haddad C., Mockenhaupt M., Dunant A., Liss Y., Bork K., Haustein U.F., Vieluf D., Roujeau J.C., Le Louet H. (2010). ALDEN, an Algorithm for Assessment of Drug Causality in Stevens–Johnson Syndrome and Toxic Epidermal Necrolysis: Comparison With Case–Control Analysis. Clin. Pharmacol. Ther..

[26] Hetherington S., McGuirk S., Powell G., Cutrell A., Naderer O., Spreen B., Lafon S., Pearce G., Steel H. (2001). Hypersensitivity reactions during therapy with the nucleoside reverse transcriptase inhibitor abacavir. Clin. Ther..

[27] Mallal S., Phillips E., Carosi G., Molina J.-M., Workman C., Tomažič J., Jägel-Guedes E., Rugina S., Kozyrev O., Cid J.F. (2008). HLA-B*5701 Screening for Hypersensitivity to Abacavir. N. Engl. J. Med..

[28] Chessman D., Kostenko L., Lethborg T., Purcell A.W., Williamson N.A., Chen Z., Kjer-Nielsen L., Mifsud N.A., Tait B.D., Holdsworth R. (2008). Human Leukocyte Antigen Class I-Restricted Activation of CD8+ T Cells Provides the Immunogenetic Basis of a Systemic Drug Hypersensitivity. Immunity.

[29] Illing P.T., Vivian J.P., Dudek N.L., Kostenko L., Chen Z., Bharadwaj M., Miles J.J., Kjer-Nielsen L., Gras S., Williamson N.A. (2012). Immune self-reactivity triggered by drug-modified HLA-peptide repertoire. Nature.

[30] Saag M., Balu R., Phillips E., Brachman P., Martorell C., Burman W., Stancil B., Mosteller M., Brothers C., Wannamaker P. (2008). High Sensitivity of Human Leukocyte Antigen–B*5701 as a Marker for Immunologically Confirmed Abacavir Hypersensitivity in White and Black Patients. Clin. Infect. Dis..

[31] Genin E., Chen D.-P., Hung S.-I., Sekula P., Schumacher M.A., Chang P.-Y., Tsai S.-H., Wu T.-L., Bellón T., Tamouza R. (2013). HLA-A*31:01 and different types of carbamazepine-induced severe cutaneous adverse reactions: An international study and meta-analysis. Pharm. J..

[32] Simper G.S., Hò G.-G., Celik A., Huyton T., Kuhn J., Kunze-Schumacher H., Blasczyk R., Bade-Döding C. (2018). Carbamazepine-Mediated Adverse Drug Reactions: CBZ-10,11-epoxide but Not Carbamazepine Induces the Alteration of Peptides Presented by HLA-B *15:02. J. Immunol. Res..

[33] Haukamp F.J., Gall E., Hò G.-G.T., Hiemisch W., Stieglitz F., Kuhn J., Blasczyk R., Pich A., Bade-Döding C. (2022). Unravelling the Proteomics of HLA-B*57:01^+^ Antigen Presenting Cells during Abacavir Medication. J. Pers. Med..

[34] Hò G.-G.T., Heinen F.J., Huyton T., Blasczyk R., Bade-Döding C. (2019). HLA-F*01:01 presents peptides with N-terminal flexibility and a preferred length of 16 residues. Immunogenetics.

[35] Gall E., Stieglitz F., Pich A., Behrens G.M.N., Kuhn J., Blasczyk R., Haukamp F.J., Bade-Döding C. (2022). Proteomic Profiling and T Cell Receptor Usage of Abacavir Susceptible Subjects. Biomedicines.

[36] Cox J., Mann M. (2008). MaxQuant enables high peptide identification rates, individualized p.p.b.-range mass accuracies and proteome-wide protein quantification. Nat. Biotechnol..

[37] UniProt C. (2021). UniProt: The universal protein knowledgebase in 2021. Nucleic Acids Res..

[38] Tyanova S., Temu T., Sinitcyn P., Carlson A., Hein M.Y., Geiger T., Mann M., Cox J. (2016). The Perseus computational platform for comprehensive analysis of (prote)omics data. Nat. Methods.

[39] R Core Team (2013). R: A Language and Environment for Statistical Computing.

[40] Gu Z., Eils R., Schlesner M. (2016). Complex heatmaps reveal patterns and correlations in multidimensional genomic data. Bioinformatics.

[41] Wickham H. (2016). ggplot2: Elegant Graphics for Data Analysis.

[42] Subramanian A., Tamayo P., Mootha V.K., Mukherjee S., Ebert B.L., Gillette M.A., Paulovich A., Pomeroy S.L., Golub T.R., Lander E.S. (2005). Gene set enrichment analysis: A knowledge-based approach for interpreting genome-wide expression profiles. Proc. Natl. Acad. Sci. USA.

[43] Mootha V.K., Lindgren C.M., Eriksson K.-F., Subramanian A., Sihag S., Lehar J., Puigserver P., Carlsson E., Ridderstråle M., Laurila E. (2003). PGC-1α-responsive genes involved in oxidative phosphorylation are coordinately downregulated in human diabetes. Nat. Genet..

[44] Griss J., Perez-Riverol Y., Lewis S., Tabb D.L., Dianes J.A., Del-Toro N., Rurik M., Walzer M., Kohlbacher O., Hermjakob H. (2016). Recognizing millions of consistently unidentified spectra across hundreds of shotgun proteomics datasets. Nat. Methods.

[45] Kunze-Schumacher H., Blasczyk R., Bade-Doeding C. (2014). Soluble HLA Technology as a Strategy to Evaluate the Impact of HLA Mismatches. J. Immunol. Res..

[46] Charlton O.A., Harris V.R., Phan K., Mewton M.E., Jackson C.J., Cooper A. (2020). Toxic Epidermal Necrolysis and Steven–Johnson Syndrome: A Comprehensive Review. Adv. Wound Care.

[47] (2009). Phase 0 trials: A platform for drug development?. Lancet.

[48] Le Tourneau C., Lee J., Siu L. (2009). Dose escalation methods in phase I cancer clinical trials. J. Natl. Cancer Inst..

[49] Kerr B.M., Thummel K.E., Wurden C.J., Klein S.M., Kroetz D.L., Gonzalez F.J., Levy R. (1994). Human liver carbamazepine metabolism: Role of CYP3A4 and CYP2C8 in 10,11-epoxide formation. Biochem. Pharmacol..

[50] Madson J.G., Lynch D.T., Tinkum K.L., Putta S.K., Hansen L.A. (2006). Erbb2 Regulates Inflammation and Proliferation in the Skin after Ultraviolet Irradiation. Am. J. Pathol..

[51] Cardinez C., Miraghazadeh B., Tanita K., Da Silva E., Hoshino A., Okada S., Chand R., Asano T., Tsumura M., Yoshida K. (2018). Gain-of-function IKBKB mutation causes human combined immune deficiency. J. Exp. Med..

[52] Snyder M., He W., Zhang J.J. (2005). The DNA replication factor MCM5 is essential for Stat1-mediated transcriptional activation. Proc. Natl. Acad. Sci. USA.

[53] Banerjee S., Biehl A., Gadina M., Hasni S., Schwartz D.M. (2017). JAK–STAT Signaling as a Target for Inflammatory and Autoimmune Diseases: Current and Future Prospects. Drugs.

[54] Fuchs J., Cheblal A., Gasser S. (2021). Underappreciated Roles of DNA Polymerase delta in Replication Stress Survival. Trends Genet..

[55] Zhang Z. (2022). POLD2 is activated by E2F1 to promote triple-negative breast cancer proliferation. Front. Oncol..

[56] Cong F., Long J., Liu J., Deng Z., Yan B., Liang C., Huang X., Liu J., Tang W. (2022). An integrative analysis revealing POLD2 as a tumor suppressive immune protein and prognostic biomarker in pan-cancer. Front. Genet..

[57] Orlandi A., Ferlosio A., Arcuri G., Scioli M.G., De Falco S., Spagnoli L.G. (2010). Flt-1 expression influences apoptotic susceptibility of vascular smooth muscle cells through the NF-kappaB/IAP-1 pathway. Cardiovasc. Res..

[58] Jackson N.M., Ceresa B.P. (2017). EGFR-mediated apoptosis via STAT3. Exp. Cell Res..

[59] Hall I.P. (2020). FLT1: A potential therapeutic target in sepsis-associated ARDS?. Lancet Respir. Med..

[60] Sabbah D.A., Hajjo R., Sweidan K. (2020). Review on Epidermal Growth Factor Receptor (EGFR) Structure, Signaling Pathways, Interactions, and Recent Updates of EGFR Inhibitors. Curr. Top. Med. Chem..

[61] Paul C., Wolkenstein P., Adle H., Wechsler J., Garchon H., Revuz J., Roujeau J. (1996). Apoptosis as a mechanism of keratinocyte death in toxic epidermal necrolysis. Br. J. Dermatol..

[62] Liu W., Chen Y., Zeng G., Yang T., Song W. (2020). INSR mediated by transcription factor KLF4 and DNA methylation ameliorates osteoarthritis progression via inactivation of JAK2/STAT3 signaling pathway. Am. J. Transl. Res..

[63] Woo M., Hakem R., Furlonger C., Hakem A., Duncan G.S., Sasaki T., Bouchard D., Lu L., Wu G.E., Paige C.J. (2003). Caspase-3 regulates cell cycle in B cells: A consequence of substrate specificity. Nat. Immunol..

[64] Seo J., Nam Y.W., Kim S., Oh D.-B., Song J. (2021). Necroptosis molecular mechanisms: Recent findings regarding novel necroptosis regulators. Exp. Mol. Med..

[65] Bertheloot D., Latz E., Franklin B.S. (2021). Necroptosis, pyroptosis and apoptosis: An intricate game of cell death. Cell. Mol. Immunol..

[66] Shen M., Lim J.M.E., Chia C., Ren E.C. (2019). CD39+ regulatory T cells modulate the immune response to carbamazepine in HLA-B*15:02 carriers. Immunobiology.

