# Supplementary material for: HLA-B*57:01/Carbamazepine-10,11-Epoxide Association Triggers Upregulation of the NFκB and JAK/STAT Pathways
Source: Cells. 2023 Feb 21;12(5):676. doi: 10.3390/cells12050676 (PMC10000580; doi:10.3390/cells12050676)
Supplement: Supplementary file 1 [file cells-12-00676-s001.zip › cells-2143909-supplementary.pdf]

Supplementary Materials

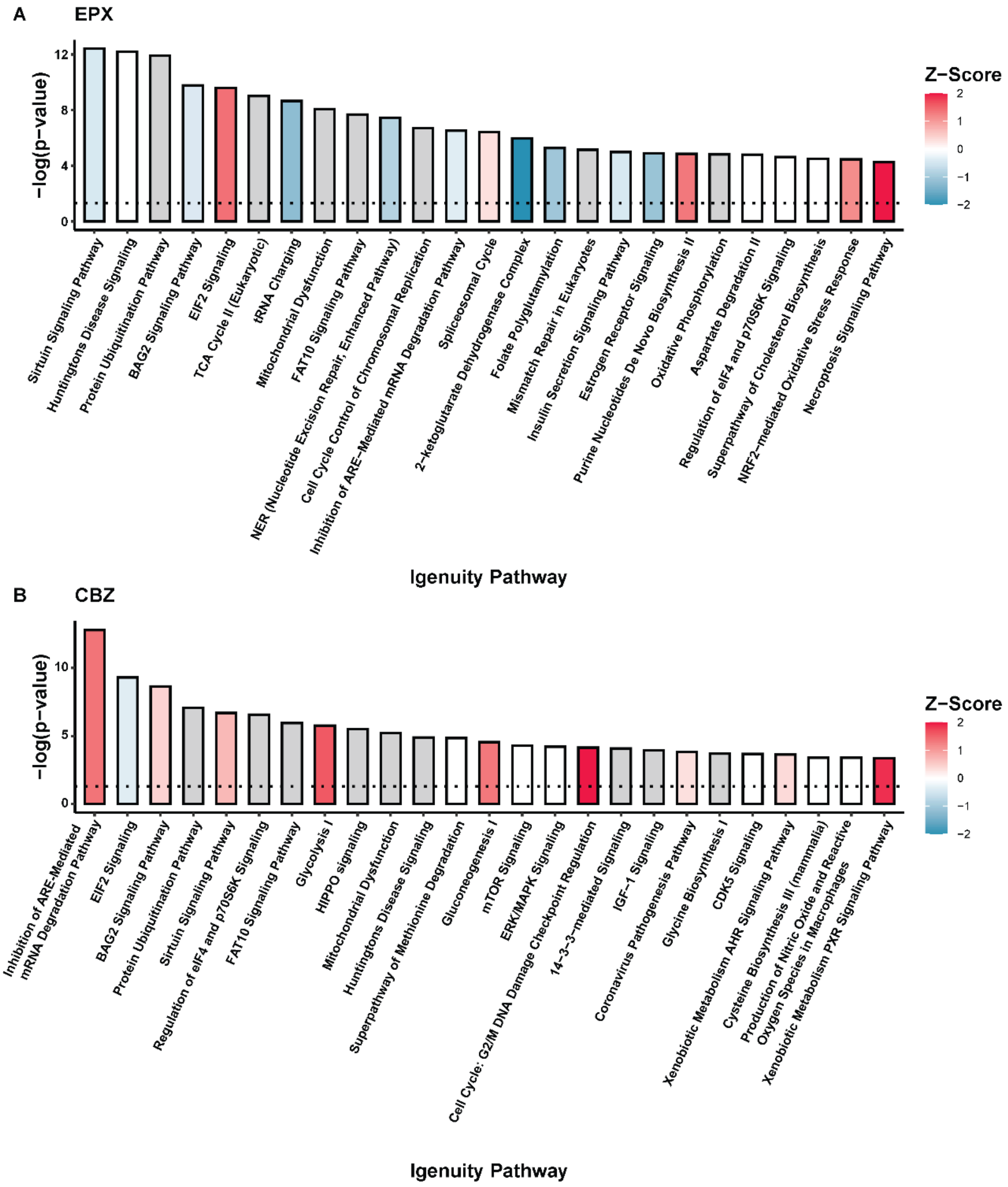

Figure S1: Ingenuity pathway analysis of proteomic changes induced by EPX or CBZ treatment in *LCL721.221/mHLA-B*57:01* cells vs parental cells.

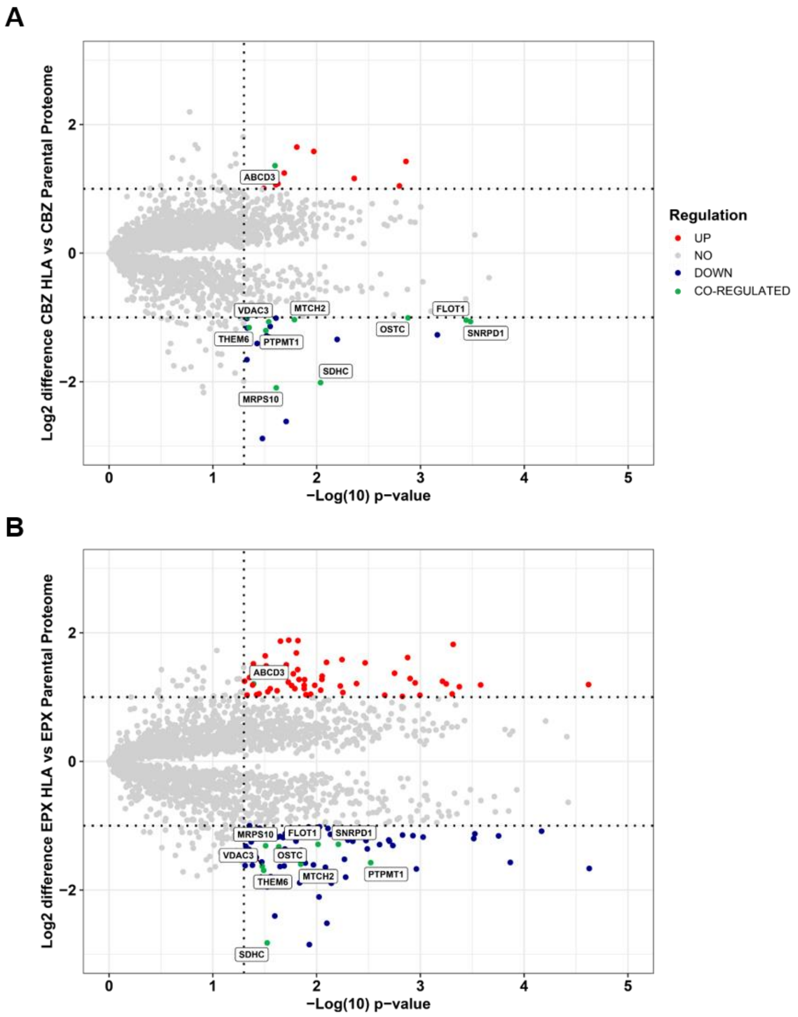

Figure S2: Analysis of proteomic changes and predicted upstream regulators after treatment of *LCL721.221/sHLA-B*57:01* and parental *LCL721.221* cells with CBZ or EPX. Volcano plot of protein expression in *LCL721.221/sHLA-B*57:01* vs. parental *LCL721.221* cells after CBZ (**A**) or EPX treatment (**B**). Labeled proteins overlap between both treatment conditions.
